# The Classification of Tongue Colors with Standardized Acquisition and ICC Profile Correction in Traditional Chinese Medicine

**DOI:** 10.1155/2016/3510807

**Published:** 2016-12-06

**Authors:** Zhen Qi, Li-ping Tu, Jing-bo Chen, Xiao-juan Hu, Jia-tuo Xu, Zhi-feng Zhang

**Affiliations:** ^1^Department of Basic Medical College, Shanghai University of Traditional Chinese Medicine, 1200 Cailun Road, Pudong New Area, Shanghai 201203, China; ^2^College of Computer Science and Technology, Jilin University, Jilin 130012, China; ^3^Shanghai Collaborative Innovation Center of Health Service in Traditional Chinese Medicine, Shanghai University of Traditional Chinese Medicine, 1200 Cailun Road, Shanghai 201203, China

## Abstract

*Background and Goal*. The application of digital image processing techniques and machine learning methods in tongue image classification in Traditional Chinese Medicine (TCM) has been widely studied nowadays. However, it is difficult for the outcomes to generalize because of lack of color reproducibility and image standardization. Our study aims at the exploration of tongue colors classification with a standardized tongue image acquisition process and color correction.* Methods*. Three traditional Chinese medical experts are chosen to identify the selected tongue pictures taken by the TDA-1 tongue imaging device in TIFF format through ICC profile correction. Then we compare the mean value of *L* ^*^ *a* ^*^ *b* ^*^ of different tongue colors and evaluate the effect of the tongue color classification by machine learning methods.* Results*. The *L* ^*^ *a* ^*^ *b* ^*^ values of the five tongue colors are statistically different. Random forest method has a better performance than SVM in classification. SMOTE algorithm can increase classification accuracy by solving the imbalance of the varied color samples.* Conclusions*. At the premise of standardized tongue acquisition and color reproduction, preliminary objectification of tongue color classification in Traditional Chinese Medicine (TCM) is feasible.

## 1. Introduction

Tongue inspection, an important diagnosis tool in Traditional Chinese Medicine (TCM), is essential for clinical syndrome differentiation and therapeutic evaluation. The traditional tongue inspection in TCM mainly focuses on the visual description other than the objective quantification and as a result limits the development of diagnostic methods and techniques in TCM to a certain extent.

However, with the development of computer techniques, application of digital image processing techniques in tongue diagnosis has been widely studied, such as segmentation of the tongue image [1–3], separation of tongue substance and tongue coating [4, 5], and analysis of tongue image [6].

Recently, on the basis of the above research results, more and more researchers attempt to use machine learning methods in tongue image recognition or classification. Their research can be grouped into four categories, shape [7–9], texture [10], color [11–15], and comprehensive feature analysis of tongue images [16, 17].

The majority pays more attention to the machine learning for the fuzziness and uncertainty of tongue images due to the uneven personal experiences of observers and different illumination conditions. According to two different color measurement systems, studies on the objective classification of tongue color are mainly implemented through the visible reflection spectrum [12, 13] and the commonly used pixelwise or color space, respectively [14, 15].

There are only a few studies on former measurement system used in the tongue color classification. Most researchers try to recognize the tongue color on the basis of the pixelwise or color space. For example, Yamamoto et al. observed that the tongue color change in different periods in the device-independent International Commission on Illumination (CIE) 1976 *L* ^*∗*^ *a* ^*∗*^ *b* ^*∗*^ color space varied between individuals [18].

On the other hand, some researchers attempt to observe the difference of the tongue color identification outcomes accompanied with different medical practitioners. Oji et al. once studied the color discrimination and tongue color diagnosis in 68 traditional Japanese medical practitioners by the Farnsworth-Munsell 100 Hue test and 84 tongue images, respectively, and they found out that overall color discrimination became worse with aging [19].

Another group of Japanese researchers had chosen 10 experienced Kampo medicine physicians to identify the tongue images acquired by the DS01-B tongue color information acquisition system. They used* K*-means clustering algorithm to quantify tongue body and color coating information and concluded that clinically important tongue color differences in Kampo medicine can be visualized by applying machine learning to tongue images that are taken under stable conditions [15].

Machine learning methods used in tongue image classification include KNN [20, 21], Naive Bayes [22], Decision Tree [22], SVM [20, 22–25], Neutral Network [26], Graphical Models [22, 27], and Principle Component Analysis (PCA) [28]. KNN and SVM are the most commonly used methods.

Although in the past researches have made significant achievements in tongue classification and identification, there are some issues left to be discussed. First, there exists some inconsistency between the identified tongue images and the actual tongue colors observed by clinical TCM practitioners. It is because color correction or reproduction on tongue images needs to be performed. Consequently, it is difficult for the identified outcomes to generalize to the clinics. Second, tongue's objective quantization parameters vary from each research institute, making it hard to communicate between institutes. Moreover, tongue images are neither normalized nor unified. It is extremely hard to compare those models of cognition on the same platform and choose the best classification scheme.

Thus, our study would focus on the standardization of tongue collection environment [29] and color correction to overcome the above issues [30].

## 2. Materials and Methods

### 2.1. Subjects of Our Study

Since there was no available data by the literature retrieval that would indicate the effective sample size to study the classification of tongue color, our study would be carried out using tongue images sampled from First People's hospital in Taicang, Shuguang hospital, and Longhua hospital affiliated to Shanghai University of Traditional Chinese Medicine during 2011 to 2014. A total of 2230 tongue photos were collected.

### 2.2. Tongue Imaging Device and Methods

**Figure 1:**
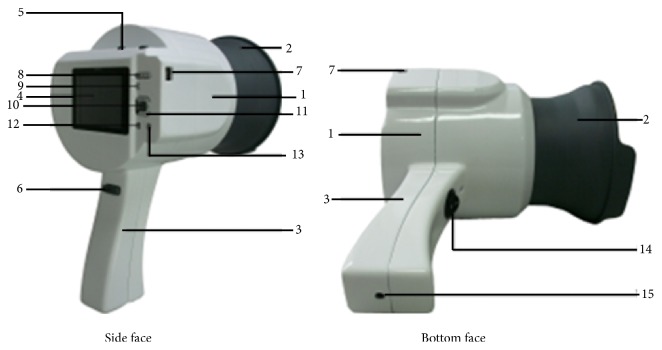
Structure chart of the TDA-1 tongue imaging device. Note: 1: telescopic cylinder, 2: removable collecting ring, 3: hand shank, 4: control panel, 5: Camera power, 6: camera button, 7: USB interface, 8: zoom button, 9: playback button, 10: OK button, 11: four-way navigation buttons, 12: MENU button, 13: SCENE button, 14: external power switch, and 15: external power connector.

TDA-1 hand-held tongue imaging device consists of image acquisition system, LED illuminator, and removable collecting ring. It is equipped with Charge Coupled Device as its photosensitive components, namely, a small-sized Eolane digital camera (Altek A12, China). The camera can capture color images with resolution of 2048 × 1536 pixels. We can manually adjust white balance, exposure time, exposure compensation, ISO speed, metering modes, flash mode, and so on. To obtain a more realistic color feature for the tongue image, LED illuminator (Kingbright KA-3021HVR4D1Z1S-C1-SH, Japan) with 6447 k color temperature, 98 color rendering index (RI), and 2413 Lux illumination is placed in the middle part of the telescopic cylinder. The whole system can create a stable light source environment for tongue image acquisition. In order to evenly illuminate the dorsal surface of the tongue, the concave reflective material is used in the back of telescopic cylinder to form a uniform irradiation light. The removable collecting ring can fix lower jaw position to ensure tongue is in a proper location in the process of tongue image collection. It can also keep out natural sunlight or unnecessary external source of light that will have disturbance on tongue and create a stable illumination environment; see Figure 1.

The operation procedure of TDA-1 tongue imaging device for tongue image collection is as follows. (1) The participants would have their mouth rinsed with water 10 minutes before imaging collection. (2) Sterilize the removable collecting ring with alcohol wipes, turn on the TDA-1 device and set up the parameters (manual mode, aperture of 13, shutter of 1/60 s, sunshine, macro lens, and nonflashlight), and then turn on the built-in light. (3) The participants would be asked to look at the front horizontally with a sitting position. And his/her jaw would be hold by the inside face of lower edge of the collecting ring which is close to the face area of the participants. After a short stabilization period of the light source (usually 5–10 s), the participants would be asked to open their mouth and protrude their tongue with a relaxed tongue body, flat tongue surface, and naturally pointing-down tongue tip. In the meantime, press the collection button when the tongue image in the preview window is appropriate. Three–five-minute rest would be needed for them if we need another collection.

### 2.3. Tongue Image Color Correction Method

In order to solve the chromatic aberration of tongue images caused by the different collection time, ICC profile correction was used in this study, and the digital ColorChecker SG was chosen. The detailed tongue image color correction based on ICC profile is as follows. First, an ICC profile is made before each batch of tongue image acquisition; the TDA-1 tongue imaging device is placed right above the digital ColorChecker SG (produced by X-rite Macbeth) in the darkroom; then take a photo of the digital ColorChecker SG and import it into the ProfileMaker software (Gretagmacbeth ProfileMaker Pro 5.0.5). Make the ICC profile of the ColorChecker SG correspond to the stable tongue acquisition light source D65. Second, import this ICC profile into Photoshop CS 4.0; the specific path is “C:∖WINDOWS∖system 32∖spool∖drivers∖color.” Import the tongue images that need color correction into Photoshop CS 4.0, assign the profile, and choose the inserted ICC profile for color correction of the original tongue images. To evaluate the effect of the ICC profile correction, we used the TDA-1 tongue imaging device to obtain 5 photos of the same SG color checker. And in each photo, 13 color-blocks (including D7, E7, F7, G7, H7, J7, D8, E8, F8, G8, H8, I8, and J8) corresponding to the human complexion would be chosen to carry out ICC profile correction and the Imatest software would be used to evaluate the color value difference (Δ*E* = [(*L*_2_^*∗*^ − *L*_1_^*∗*^)^2^ + (*a*_2_^*∗*^ − *a*_1_^*∗*^)^2^ + (*b*_2_^*∗*^ − *b*_1_^*∗*^)^2^]^1/2^).

### 2.4. Tongue Image Selection Method

**Figure 2:**
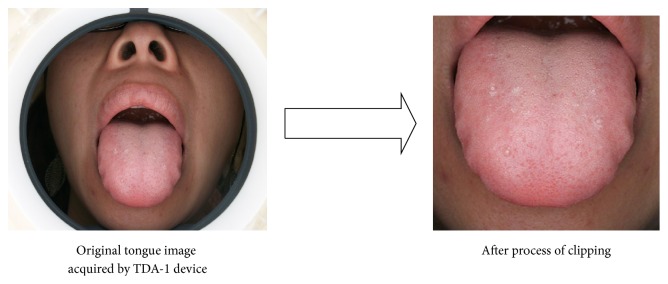


First, the tongue body area of each collected tongue photos was extracted as a size of 300 × 300 in order to make the clipped tongue image close to the actual size of the tongue in clinics, and this operation was implemented by the function of tongue extraction in the software “TCM Tongue Diagnosis and Analysis System (TDAS) V2.0” (developed by our experiment team). The picture which mainly included the tongue body in its middle part was manually framed in a square, and the clipped tongue image would be automatically saved as a size of 300 × 300 by clicking the save function (Figure 2). Then look through those extracted tongue body images on the same computer monitors (Lenovo AIO has resolution of 1440 × 900); tongue pictures would be excluded if they fail to satisfy the requirements due to wrong position of tongue protrusion, underexposure or overexposure, blur, invisible tongue body covered by tongue coating, and so on. Tongue images which had petechia or ecchymosis or prick or barb on the tongue surface would also be discarded since they can be disturbance in color recognition of tongue surface.

### 2.5. Tongue Image Identification Method

The color of tongue images in TIFF format after color correction would be recognized by three TCM diagnosis professionals. Those that received the agreement from all three professionals would be included in this study. According to the literature's reporting, tongue color would be classified into five groups, namely, pale tongue, light red tongue, red tongue, crimson tongue, and purplish tongue (including light purple tongue and blue and purple tongue) [15, 19].

### 2.6. The Selection and Analysis of Characteristic Parameter for the Color of Tongue Images

**Figure 3:**
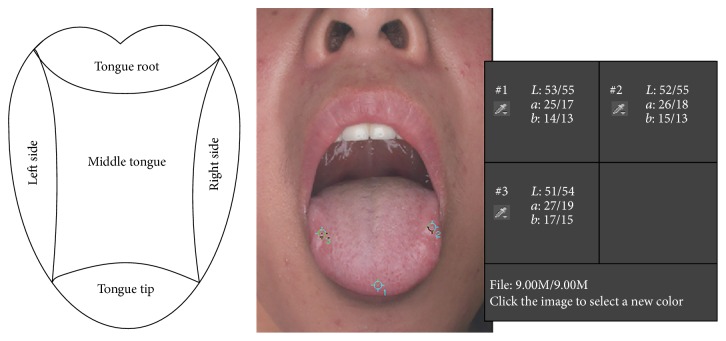


Given the conformance and intuition between different color space's description on tongue color and the regular TCM diagnostic description for tongue color, *L* ^*∗*^ *a* ^*∗*^ *b* ^*∗*^ color space would be used in this study. The *L* ^*∗*^ value represents the luminosity, which means the lightness of color, ranging from 0 to 100 (absolute black to absolute white). The *a* ^*∗*^ value runs in range of +127 to −128 (red to green) and the *b* ^*∗*^ value suggests the range from yellow to blue with the value between +127 and −128 (yellow to blue). Besides, in order to avoid the variation in tongue color classification index caused by different image segmentation techniques, the mean value of *L* ^*∗*^, *a* ^*∗*^, and *b* ^*∗*^ before or after the color correction would be acquired by manual selection of tongue tip, tongue left side, and tongue right side in each tongue image with the color sampler tool of 5 × 5 mm in Photoshop CS 4.0 (Figure 3).

### 2.7. Statistical Method and Data Mining Method

*t*-test would be used in the comparison of Δ*E* before and after the ICC color correction. Dunnett's T3 multiple comparison test in ANOVA would be used for the pairwise comparison of *L* ^*∗*^, *a* ^*∗*^, and *b* ^*∗*^ value of different color groups in SPSS18.0. Consider the idea that SVM is the most commonly used supervised machine learning method in tongue diagnosis [31], while Random forest is rarely used in this area according to the reports; besides the tongue color classification in this study is a multiclassification research; meanwhile the samples are limited and imbalanced, and the feature number for the studied images is relatively small; in order to obtain a better classification accuracy, we will use SVM and Random forest in the modeling and analytical test for the experimental data in WEKA software [32].

## 3. Results

### 3.1. Results for the Evaluation of ICC Profile Correction

The color value difference (Δ*E*) of the 65 color-blocks in the SG color checker before ICC profile correction is 11.79 ± 1.67, it would decrease to 4.50 ± 2.41 after ICC profile correction, and it was statistically evident between them (*p* < 0.05). As we know, the value of Δ*E* indicates the total difference of varied colors, and a bigger Δ*E* value means a larger color difference. Generally speaking, when Δ*E* is more than 12.0, it indicates that the two colors are entirely different; while it is less than 1.5, the difference is beyond the human vision's sensation [33]. Therefore, from the above outcomes, the color difference is obviously reduced after the ICC profile correction.

### 3.2. Tongue Color's Distribution in the *L* ^*∗*^ *a* ^*∗*^ *b* ^*∗*^ Color Space before and after the Correction

**Figure 4:**
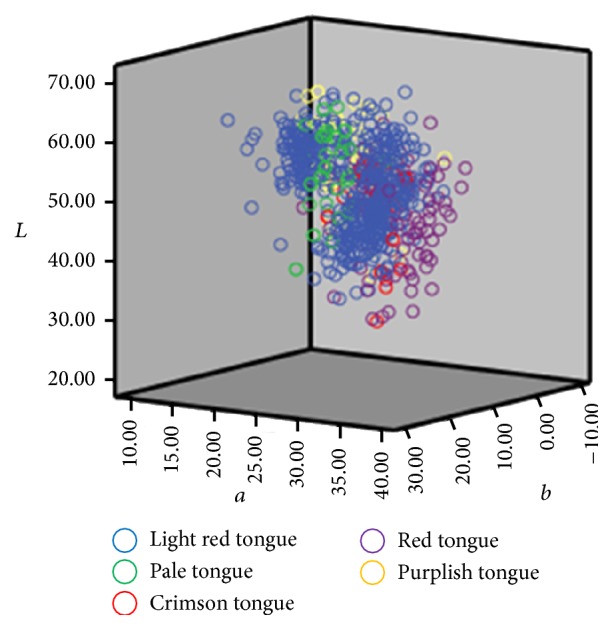
*L* ^*∗*^ *a* ^*∗*^ *b* ^*∗*^ color space distribution for varied tongue colors (before color correction).

**Figure 5:**
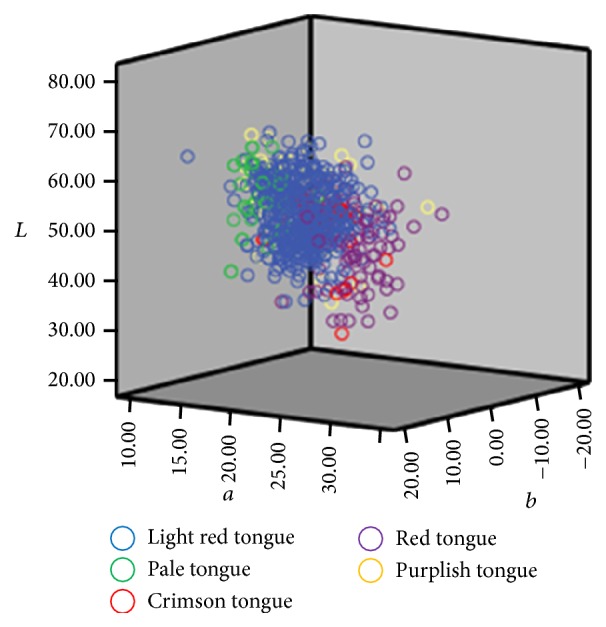
*L* ^*∗*^ *a* ^*∗*^ *b* ^*∗*^ color space distribution for varied tongue colors (after color correction).

Tongue color's distribution in the *L* ^*∗*^ *a* ^*∗*^ *b* ^*∗*^ color space before and after the correction by ICC profile was demonstrated in Figures 4 and 5. Those figures indicate that the total dispersion of tongue color was reduced after correction, especially for the light red tongue's distribution in the *L* ^*∗*^ *a* ^*∗*^ *b* ^*∗*^ color space. It means that the tongue colors have a more centralized and balanced distribution in the *L* ^*∗*^ *a* ^*∗*^ *b* ^*∗*^ color space, so that it would be more beneficial for the TCM practitioners to recognize.

### 3.3. The Comparison for the Feature Parameter of Intergroup Tongue Colors

The comparison result for different tongue colors is shown in Table 1.

The results in Table 1 indicate the following aspects. (1) As for the *L* ^*∗*^ value, most colors have a statistical significance in their pairwise comparison (*p* < 0.05) except for the comparison between purplish tongue and light red tongue/pale tongue as well as the comparison between the red tongue and crimson tongue. For the *L* ^*∗*^ value suggesting the lightness of a certain color, from Table 1 we could conclude that the lightness of the red or crimson tongue is relatively lower than other three colors, and the light red tongue has lower lightness than the pale tongue. There was no obvious difference in lightness between the purplish tongue and the light red tongue/the pale tongue, and so did the red tongue versus the crimson tongue. (2) As for the *a* ^*∗*^ value, except for the light red tongue versus the crimson tongue and the pale tongue versus the purplish tongue, the rest colors are statistically evident for their pairwise comparison. And the *a* ^*∗*^ mean value of different tongue colors shows that the red tongue had the highest red component, next is light red tongue/crimson tongue, and pale tongue/purplish tongue is the lowest in the red component, while no obvious difference was found in both pale tongue versus purplish tongue and light red tongue versus crimson tongue for the red component. (3) As for the *b* ^*∗*^ value, each tongue color is statistically significant (*p* < 0.05) with the rest of tongue colors except the light red tongue versus the pale tongue, and the mean value of *b* ^*∗*^ of those varied colors shows that the blue component was decreased from purplish tongue to crimson tongue and then to red tongue. We can also tell that light red tongue or pale tongue has the lowest blue component and there is no obvious difference between them.

**Figure 6:**
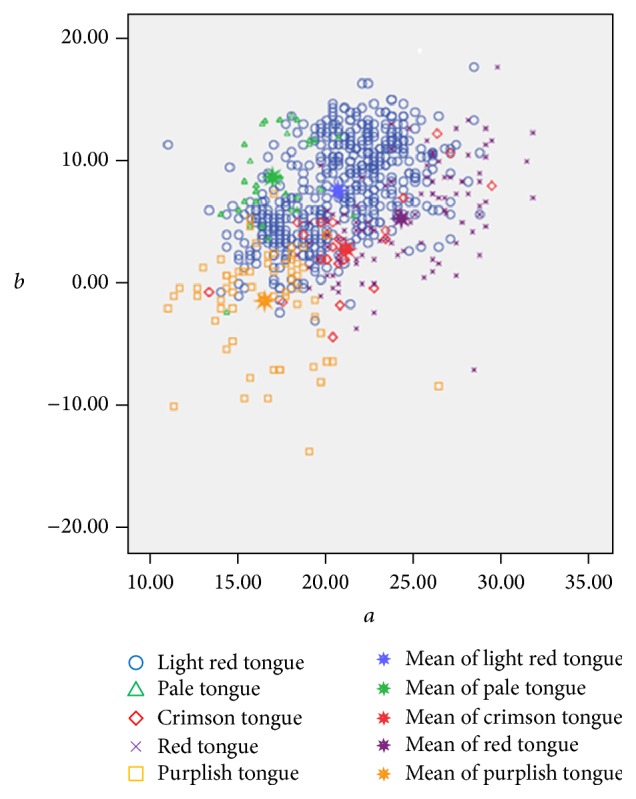
Distribution of respective average tongue colors in *a* ^*∗*^ *b* ^*∗*^ plane color space. Note: (1) *a* ^*∗*^ value: purplish tongue < pale tongue < light red tongue < crimson tongue < red tongue, which indicates that the red components are decreasing. (2) *b* ^*∗*^ value: purplish tongue < crimson tongue < red tongue < light red tongue < pale tongue, which indicates that the blue components are decreasing, while the yellow component is increasing.

The results in Table 1 indicate that the difference of those five tongue colors is more obvious in *a* ^*∗*^ and *b* ^*∗*^ value. The distribution of different tongue colors in *a* ^*∗*^ *b* ^*∗*^ plane was demonstrated in Figure 6. Even though there exist certain overlaps at the boundaries between some colors in the *a* ^*∗*^ *b* ^*∗*^ plane color space distribution due to the absence of *L* ^*∗*^ value, general tendency can be found in the distribution of varied tongue colors in the *a* ^*∗*^ *b* ^*∗*^ plane color space, namely, from the pale tongue to the light red tongue and then to the red tongue; their color is gradually changed. In other words, it is a progress of gradual increase of *a* ^*∗*^ value and gradual decrease of *b* ^*∗*^ value, which indicates the gradual increase of red component and gradual decrease of yellow components in their colors. Besides, the purplish tongue has both the lowest *a* ^*∗*^ value and the lowest *b* ^*∗*^ value (negative value), which indicates that both the red component and yellow component of this tongue color are reduced while its blue component is augmented. For the crimson tongue, its *a* ^*∗*^ value ranks only second to the red tongue's and its *b* ^*∗*^ value is next to the lowest (purplish tongue), which suggests a relatively higher red component and a relatively lower yellow component among the five tongue colors.

### 3.4. The Results for Data Mining Modeling

WEKA software was used in the data mining modeling. The selected 728 tongue images would be studied, which included 478 cases of light red tongue, 45 cases of pale tongue, 35 cases of crimson tongue, 107 cases of red tongue, and 63 cases of purplish tongue. All original data would be processed in the “.ARFF” format under the software's requirement.

In order to fulfill the evaluation of effectiveness for the established model, the studied tongue images would be divided into two groups by 10-fold cross-validation: one is the training set for the training of the best model and the other is testing set for the evaluation of the classification effect. In order to solve the problem of imbalance of tongue color samples, SMOTE algorithm would be used in this study to the sample amplification of 2390 cases. Furthermore, SVM and Random forest would be applied, respectively, for analytical test and the evaluation of the classification accuracy of model.

The results are shown in Tables 2, 3, 4, 5, and 6.  Table 2 demonstrates that the overall classification accuracy for tongue color is relatively high. Random forest has a higher classification accuracy than LibSVM either before or after the sample amplification. The classification accuracy of both Random forest and SVM is increased after the sample amplification. The accuracy of SVM increases from 74.59% to 79.83%, while the accuracy of Random forest increases from 78.71% to 84.94%.

Furthermore, the classification accuracy of SVM and Random forest for different tongue colors was shown in Table 7. We can see that, without sample amplification, Random forest achieved relatively higher classification accuracy for varied tongue colors than SVM. However, after sample amplification both SVM and Random forest's classification accuracy for the light red tongue were decreased, but the whole classification accuracy for the rest tongue colors is obviously improved.

### 3.5. Diagnostic Value for Different Classifiers

Tongue weighted average area under the ROC curve (Auc) would be calculated for both SVM and Random forest (Table 8). The Auc for both classifiers is approximately between 0.69 and 0.98 whether before or after the amplification. This is a fairly good diagnostic value in terms of the diagnostic significance of area. And the Auc of the two classified models is 0.69 and 0.89, respectively, before the amplification but increases to 0.87 and 0.98 after the amplification. Therefore, the Auc of the Random forest is higher than LibSVM no matter whether the samples were amplified or not. Besides, the Auc of the two classification model is largely increased after the sample amplification. Combined with the accuracy above, it can be concluded that the Random forest has a better established classification model for the tongue, and the sample amplification can solve the imbalance of different tongue colors effectively. Therefore, our method has great diagnostic significance in clinics.

## 4. Discussion

Along with the achievements obtained in the digitization and objectification of tongue image diagnosis in TCM, many other researches about prediction of health state or abnormity of physical indicators in western medicine have been done recently [20, 22–25, 28, 34, 35]. Though they have successfully set up certain connection between the objective tongue feature parameter and the health state, it is difficult for them to be accepted and admitted by the TCM clinicians for their abstraction in the tongue diagnosis. Therefore, our study successfully establishes the connection between the visualized tongue color diagnosed by TCM experts and the abstract objective feature parameters through the exploration on the classification of different tongue colors in TCM.

Tongue color, generally, is clinically classified into categories as pale, light red, red, crimson, blue, purple, and others. For there is no strict boundaries among them, the color recognition of each observer is also varied. In this study, tongue color is classified into light red tongue, pale tongue, red tongue, crimson tongue, and purplish tongue on the basis of relevant literature report.

ICC profile is a kind of color management method based on the ICC standard by the International Color Consortium. Its basic idea is to choose a reference color space which has nothing to do with the equipment to describe the equipment's characteristic; therefore, it can make it up to the loss of color information caused by equipment. So far, this color management method has been admitted by a lot of operating systems and equipment manufacturers, and it has become an internationally recognized method for color correction. From this study, it can be concluded that ICC profile correction could not only decrease color value difference (Δ*E*) evidently but also reduce the dispersion of different tongue colors effectively, which would make the identified tongue images more consistent on the coherence and authenticity with the images seen in the clinic by the TCM practitioners.

Moreover, the outcomes indicate the *a* ^*∗*^ value of the light red tongue was between the pale/purplish tongue and the red tongue (*p* < 0.05), while its *b* ^*∗*^ value was obviously higher than the red tongue, crimson tongue, and purplish tongue (*p* < 0.05). Preliminarily objective quantification could be carried out for the light red tongue in the future. As for the pale tongue, *a* ^*∗*^ value is significantly lower than the red tongue and the light red/crimson tongue (*p* < 0.05), and it has a higher *b* ^*∗*^ value than the red tongue, crimson tongue, and purplish tongue (*p* < 0.05); combined with its higher *L* ^*∗*^ value than the light red tongue (*p* < 0.05), the red component of the pale tongue is significantly decreased, corresponding to the general understanding of Traditional Chinese Medicine. The red tongue has the highest *a* ^*∗*^ value than the rest of the four colors (*p* < 0.05) and a lower *L* ^*∗*^ value than the light red tongue, pale tongue, and purplish tongue (*p* < 0.05); that is to say, its red component is relatively higher. With the red color being deeper, the brightness dimmed. As for the crimson tongue, its *L* ^*∗*^ value was obviously lower than the light red tongue, pale tongue, and purplish tongue (*p* < 0.05), its *a* ^*∗*^ value was between pale/purplish tongue and red tongue (*p* < 0.05), and its *b* ^*∗*^ value is obviously lower than red tongue, light red tongue, and pale tongue (*p* < 0.05). The crimson tongue had a mediate red component but relatively small brightness. For the purplish tongue, its *a* ^*∗*^ value is obviously lower than the light red tongue, red tongue, and crimson tongue (*p* < 0.05), and its *b* ^*∗*^ value is the lowest compared to the other four colors (*p* < 0.05). This shows the decreasing of the red component and the yellow component and the tendency to be blue and purple. Totally, *a* ^*∗*^ value became gradually smaller from the red tongue to the crimson/light red tongue and to the pale/purplish tongue, which brought the decreasing of the red component. And the *L* ^*∗*^ value became gradually smaller from the pale tongue to light red tongue, to purplish tongue, and to red tongue/crimson tongue, which caused the gradually dim brightness. As for the *b* ^*∗*^ value, the purplish tongue was the lowest compared to the others, which indicated its highest blue component.

From the perspective of *L* ^*∗*^ *a* ^*∗*^ *b* ^*∗*^ color space, the main characteristics index of pale tongue and red tongue were *L* ^*∗*^ and *a* ^*∗*^ value; namely, the pale tongue had a higher *L* ^*∗*^ value and a lower *a* ^*∗*^ value than normal tongue color, while the red tongue is the opposite. The main characteristics index of the crimson tongue is lower *L* ^*∗*^ value than the normal tongue color. And the purplish tongue shows the lowest *b* ^*∗*^ value and the decreasing of *a* ^*∗*^ value.

This experiment suggests the different characteristics in *L* ^*∗*^ *a* ^*∗*^ *b* ^*∗*^ color space for varied colors. For the imbalance and limitation of the studied samples, some index of the five tongue color in the *L* ^*∗*^ *a* ^*∗*^ *b* ^*∗*^ color space has shown no significant difference, such as the *L* ^*∗*^ value for the red and crimson tongue, *a* ^*∗*^ value of the pale tongue and purplish tongue, *a* ^*∗*^ value of the light red tongue and crimson tongue, and *b* ^*∗*^ value of the light red tongue and pale tongue. Therefore, it is feasible for the standardization and formulation of medical reference range for tongue colors in TCM and it is necessary to increase the sample size of other abnormal tongue colors in the further study.

In addition, we also study the tongue color classification in the application of machine learning methods. Comparing to the commonly used SVM algorithm, Random forest has a better result on the tongue color classification despite using imbalanced sample for different tongue colors (Tables 2, 7, and 8). Moreover, SMOTE algorithm could improve both the whole accuracy of tongue color classification and the accuracy of those abnormal tongue color classification effectively by sample amplification (Tables 2, 3, 4, 5, 6, 7, and 8). Consistency of the tongue image collection process and the use of the color correction ensured the uniformity and authenticity of the tongue pictures' quality. The inconformity of tongue image segmentation during the tongue image processing procedure would lead to the difference of tongue color classification index, so an agreement should be reached in the future on the tongue image processing method. Only 3 experts are chosen to recognize the tongue color in this study; on account of the difference of the subjective experience of varied observers and the uncertainty of the classification standard, the consensus on the TCM expert diagnosis system of the tongue images should be reached in the future.

Last but not least, color is a low-level feature for tongue images. The identification and extraction of other tongue features like texture or fissures are based on the color information of the digital tongue image. Therefore, we initially researched the objective classification of tongue color. Given the idea that TCM normally combines color, texture, and fissures for diagnosis rather than the single factor color, studies on the objective and quantitative classification of texture and fissures are still required in the future.

## 5. Conclusions

In this study, preliminary objectification of tongue color classification is feasible on the basis of standardized tongue acquisition and color correction. Data mining methods of SVM and Random forest can help us to evaluate the classification results effectively. Random forest has a better performance in the classification accuracy of abnormal tongue colors than SVM. SMOTE algorithm can improve the classification accuracy by solving the imbalance of the studied samples. Our research would contribute to the automatic diagnostic system of tongue image in TCM.

## Figures and Tables

**Table 1:** Comparison for the different tongue color's *L* ^*∗*^,  *a* ^*∗*^, and  *b* ^*∗*^ value ($\overset{-}{x}\pm s$).

Group	Number (*n* = 729)	*L* ^*∗*^	*a* ^*∗*^	*b* ^*∗*^
Light red	478	53.31 ± 5.90^▲★*∗*^	20.63 ± 3.08^▲*∗*■^	7.6 ± 3.93^★*∗*■^
Pale	45	56.18 ± 6.06^●★*∗*^	16.93 ± 1.75^●★*∗*^	8.69 ± 3.48^★*∗*■^
Crimson	35	48.07 ± 6.12^●▲■^	21.08 ± 3.14^▲*∗*■^	2.83 ± 3.65^●▲*∗*■^
Red	107	48.43 ± 6.57^●▲■^	24.21 ± 3.43^●▲★■^	5.32 ± 4.14^●▲★■^
purplish	63	52.46 ± 6.53^★*∗*^	16.47 ± 2.67^●★*∗*^	−1.39 ± 4.22^●▲★*∗*^

^●^Compared with light red tongue *p* < 0.05.

^★^Compared with crimson tongue *p* < 0.05.

^■^Compared with purplish tongue *p* < 0.05.

^▲^Compared with pale tongue *p* < 0.05.

^*∗*^Compared with red tongue *p* < 0.05.

**Table 2:** Comparison of classification accuracy.

Classification accuracy (%)	samples	LibSVM	Random forest
Before sample amplification	728	74.59	78.81
After sample amplification	2390	79.83	84.94

**Table 3:** Classification results of LibSVM before amplification.

	Pale	Light red	Crimson	Red	Purplish
Pale	**14**	30	0	0	1
Light red	6	**450**	0	15	7
Crimson	0	20	**2**	11	2
Red	0	57	3	**47**	0
Purplish	1	29	2	1	**30**

**Table 4:** Classification results of LibSVM after amplification.

	Pale	Light red	Crimson	Red	Purplish
Pale	**444**	21	1	3	9
Light red	75	**284**	26	46	47
Crimson	0	7	**404**	56	11
Red	0	21	113	**337**	7
Purplish	8	16	10	5	**439**

**Table 5:** Classification results of Random forest before amplification.

	Pale	Light red	Crimson	Red	Purplish
Pale	**32**	11	0	0	2
Light red	12	**420**	3	28	15
Crimson	0	4	**24**	5	2
Red	0	35	7	**62**	3
Purplish	1	23	2	2	**35**

**Table 6:** Classification results of Random forest after amplification.

	Pale	Light red	Crimson	Red	Purplish
Pale	**439**	30	0	1	8
Light red	41	**338**	21	43	35
Crimson	0	13	**408**	46	11
Red	0	28	45	**399**	6
Purplish	4	18	6	4	**446**

**Table 7:** Classification accuracy of different tongue colors (%).

Amplification	LibSVM		Random forest	
	NO	YES	NO	YES
Pale	31.1	92.9	71.1	91.8
Light red	94.1	59.4	87.9	70.7
Crimson	5.7	84.5	68.6	85.3
Red	43.9	70.5	57.9	83.5
Purplish	47.6	91.8	55.5	93.3

**Table 8:** Auc for different machine learning methods.

Auc	LibSVM	Random forest
Before amplification	0.69	0.89
After amplification	0.87	0.98
